# Can the diameter of nerve fibers be effectively utilized to enhance the accuracy of determining the length of the aganglionic segment compared to intraoperative biopsy in patients with Hirschsprung’s disease?

**DOI:** 10.1186/s13104-024-06873-x

**Published:** 2024-08-06

**Authors:** Ali Talebi, Mohammadsadegh Talebi Kahdouei, Elmira Hajiesmaeil Memar, Bahar Ashjaei, Hojatollah Raji, Maryam Ghavami Adel

**Affiliations:** 1 Pediatric Disease Research Center, Guilan University of Medical Science, Rasht, Iran; 2 Medical school, Tehran University of Medical Science, Tehran, Iran; 3 Children’s Medical Center, Tehran University of Medical Science, Tehran, Iran; 4 Department of Pediatric Surgery, Children’s Medical Center, Tehran University of Medical Science, Tehran, Iran

**Keywords:** Hirschsprung, Neural fiber trunk diameter, Aganglionic segment, Biopsy

## Abstract

**Objective:**

The aim of this study is to investigate the accuracy of utilizing neural fiber trunk diameter in accurately diagnosing the length of the aganglionic segment in patients definitively diagnosed with Hirschsprung’s disease.

**Results:**

In this study, 40 patients (19 males, 21 females; mean age 2.5 ± 2.2646 years) were assessed for Hirschsprung’s disease. Constipation was the main symptom (75%), followed by abdominal issues. All underwent contrast enema and rectal suction biopsy for diagnosis, followed by surgery (predominantly Soave and Swensen techniques). Majority (85%) had rectosigmoid involvement. Neural fiber diameter was measured, with 52.5% ≤40 μm and 47.5% >40 μm. Statistical analysis showed 40% sensitivity(CI:95%) and 47% specificity(CI:95%) with a cutoff of 40.5 μm. Cohen’s kappa index for aganglionic segment size was 0.7.

## Introduction

Hirschsprung’s disease is a congenital disorder of the intestines, occurring in approximately 1 in every 5000 births. Its characteristic feature is the absence of ganglion cells in the myenteric plexus and submucosal plexus in a segment of the intestine [1]. For screening patients suspected of Hirschsprung’s disease, contrast enema is initially performed. If the contrast enema shows suspicious findings, the patient undergoes biopsy, which can be done through two methods: rectal suction biopsy and full-thickness rectal biopsy. Biopsy remains the gold standard for diagnosing Hirschsprung’s disease [2]. Recent studies have shown that although contrast enema and biopsy are considered the gold standard for diagnosing Hirschsprung’s disease, they may not provide sufficient accuracy in determining the length of the aganglionic segment. As a result, it is challenging to accurately determine the length of the aganglionic segment using these methods [3]. Given that a percentage of patients still retain a portion of the aganglionic segment after surgery at the Tehran Children’s Medical Center, Our aim in conducting this study was to utilize the neural fiber trunk diameter for a more precise determination of the aganglionic segment length compared to biopsy during surgery. This was to achieve a more accurate diagnosis by precisely determining the diameter of the aganglionic segment.

## Methods and material

In this cross-sectional study, a total of 46 patients presenting to the Tehran Children’s Medical Center between 2012 and 2018 with clinical suspicion of Hirschsprung’s disease were enrolled. After ruling out other potential causes, contrast enema was performed for these patients, and rectal suction biopsy was carried out for those with suspicion, aiming to confirm Hirschsprung’s disease. Among these patients, 6 were excluded due to having a short segment aganglionic zone, leaving 40 patients meeting the study criteria. Patients were categorized into two groups based on previous studies and the length of the involved segment in biopsy specimens and contrast enemas: rectosigmoid (≥ 7 cm or ≤ 20 cm) and long segment (> 20 cm) [4]. Data including patient demographics, screening methods, surgical procedures, operative durations, length of the aganglionic segment, diameter of the nerve trunk in the affected area, intraoperation pathological finding, and relevant notes were recorded in the electronic medical records. All of these patients parent who visited the Tehran Children’s Medical Center for participation in research projects have provided written consent for their involvement.

### Biopsy methods

Biopsies were performed on these patients using the full thickness rectal biopsy method (submucosal plus muscularis propria), during which biopsies were taken from the distal and proximal regions of the area during surgery.

### Histopathological methods

Samples collected during surgery are sent to an experienced pathologist. The pathologist determines the diameter of the neural fiber trunk trunks and compares it with the length of the aganglionic segment. Ultimately, the accuracy of using the nerve trunk diameter to determine the length of the aganglionic segment is assessed. For the purpose of the pathology study, the pathologist stained the samples using the hematoxylin and eosin (H&E) method, and determined both the length of the aganglionic segment and the diameter of the nerve trunks.

### Inclusion criteria

definitive diagnosis of Hirschsprung’s disease confirmed by surgery, absence of underlying medical conditions.

### Exclusion criteria

presence of deficiencies in electronic medical records, short segment aganglionic zone, presence of underlying medical conditions.

### Data analysis

For the statistical analysis in this study, IBM SPSS version 27 software was utilized. Initially, the mean and standard deviations of the data were calculated to examine and compare the statistical data. Subsequently, the Pearson correlation test was employed to investigate the relationships among these data. Additionally, to assess the accuracy of using the diameter of nerve fibers, specificity, sensitivity, and the area under the curve (AUC) were calculated. Furthermore, the ROC curve was plotted using the same software to support this analysis.

## Result

In the current study, 40 patients (19 males and 21 females) with a mean age of 2.5 ± 2.2646 (max: 1month, min: 11month) years were included. The predominant clinical symptom among these patients was constipation, reported by 30 individuals (75%). Subsequently, 12% presented with abdominal distension and vomiting, 10% with constipation accompanied by abdominal distension, 2% with constipation and vomiting, and 1% with abdominal distension alone.

Following the exclusion of other causes, all patients underwent contrast enema screening to rule out Hirschsprung’s disease. Rectal suction biopsy was then performed for definitive confirmation of Hirschsprung’s disease. Upon confirmation, surgical intervention was undertaken. At the Tehran Pediatric Medical Center, pull-through surgery was predominantly performed using the Soave and Swensen techniques. 34 patients underwent one-stage pull-through surgery and 6 patients underwent two-stage pull-through surgery based on clinical symptoms and contrast enema findings, with 60% (24 patients) undergoing Swensen’s procedure and 40% (16 patients) undergoing Soave’s procedure. The mean duration of surgery was 2.75 ± 0.50637.

Regarding the length of the aganglionic segment, 34 patients (85%) had rectosigmoid involvement, while 6 patients (15%) had long segment aganglionic. Additionally, the diameter of the neural fiber was measured and categorized into two groups: ≤40 micrometers and > 40 micrometers. Among the patients, 52.5% had a diameter ≤ 40 micrometers, while 47.5% had a diameter > 40 micrometers. Furthermore, the mean diameter of the neural fiber was 38.6750 ± 14.33015 micrometers.(Table 1).

**Fig. 1:**
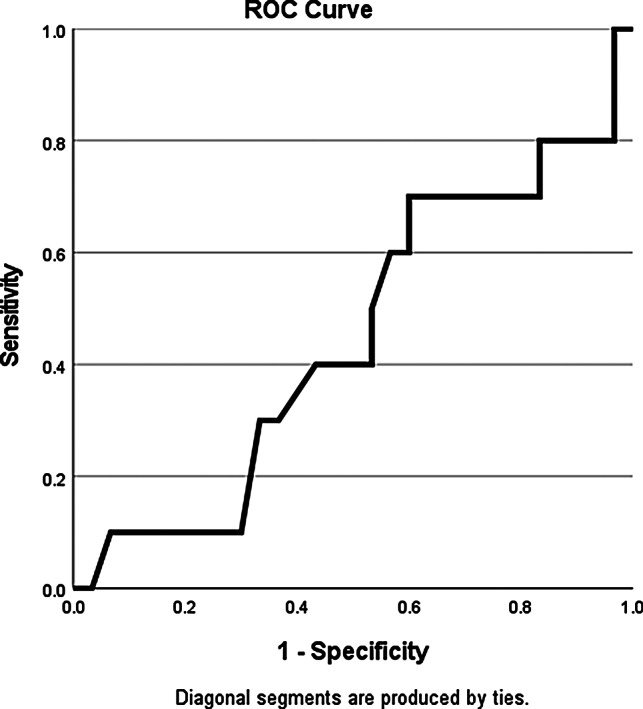
ROC curve of neural fiber trunk diameter (talebi, et al., * 2024*)

In this study, the accuracy of using the diameter of neural fiber trunks was also subjected to statistical analysis. The sensitivity and specificity of this method were 40%(CI:95%) and 47%(CI:95%) with 40.5 μm cutoff, respectively, with an AUC = 0.447.(Fig. 1) furthermore, when compared to the size determined by pathology for the aganglionic segment, the Cohen’s kappa index was found to be 0.7.(tables 2, 3).

**Table 1:** Patient’s and surgery characteristics (talebi, et al.,* 2024*)

	mean ± SD	Frequency
Age	2.5 ± 2.2646	
Gender Male(*n* = 19) Female(*n* = 21)		47.5% 52.5%
Surgical charactristics 1-stage(*n* = 34) 2-stage(*n* = 6) Surgical methods Swensen(*n* = 24) Soave (*n* = 16)		85% 15% 60% 40%
Operation duration(hour)	2.75 ± 0.50637	
Neural fiber trunks diameter ≤40 μm >40 μm	38.6750 ± 14.33015	52.5% 47.5%
Intraoperation pathological finding Rectosigmoid(≥ 7 cm or ≤ 20 cm)(*n* = 34) Long segment(> 20 cm)(*n* = 6)		85% 15%

**Table 2:** Neural fiber trunk diameter accuracy to diagnosis aganglionic zone (talebi, et al., * 2024*)

	AUC	Sensitivity		Specificity				Cohen’s kappa index
Neural fiber trunks diameter > 40 μm ≤ 40 μm	0.447	40%		47%				0.7(p-value < 0.001)

**Table 3:** Accuracy of neural fiber trunk diameter (talebi, et al., * 2024*)

	Intraoperation full-thickness biopsy		
	Rectosigmoid	Long segment	
**Neural fiber trunk diameter(> 40 μm)** Rectosigmoid Long segment	29 5 34	1 5 6	30 10 40

## Discussion

It has been established that there are various methods available for determining the length of the aganglionic segment in Hirschsprung’s disease. These methods include contrast enema and intraoperative biopsy [5]. Initially, we will review previous research regarding the utility of contrast enema. Subsequently, we will examine the precision of intraoperative biopsy. Finally, in this study, we will assess the accuracy of utilizing neural fiber trunk trunk diameter compared to intraoperative biopsy.

Previous findings regarding the localization of transition zone in Hirschsprung’s disease based on contrast enema findings and its confirmation by biopsy have shown inconsistencies. In one study investigating the performance of contrast enema in localizing the transition zone, it was found that contrast enema had an accuracy of only 30.8% in diagnosing this region [6]. Furthermoreas mentioned in some studies, the use of contrast enema in determining the location of the transition zone lacks significant accuracy. In another study, the sensitivity of contrast enema in diagnosing the transition zone was 70%, with an average specificity of 83% [7]. In the previous study conducted, the accuracy of measuring the diameter of neural fiber trunks in determining the length of the aganglionic segment was found to be low. The sensitivity of this method was 50%, with a specificity of 50% [3]. In another study, the accuracy of biopsy during surgery was examined and compared with rectal suction biopsy (RSB). The findings revealed that the accuracy of biopsy during surgery and its agreement with RSB is 98.7% [8]. In another study comparing the use of rectal suction biopsy and incisional biopsy, it was shown that incisional biopsy performs significantly better in children aged 6 months and older (24.1% vs. 0.9%, *p* < 0.01). Consequently, the study recommends using incisional biopsy over rectal suction biopsy for children in this age group [9]. In one of the conducted studies, the sensitivity and specificity of using biopsy during surgery were determined to be 100% and 100%, respectively [8]. In another study that has been conducted, the sensitivity, specificity, and accuracy of using intraoperative biopsy were 94%, 100%, and 80%, respectively [10]. In the current study, the investigation focuses on the utility of measuring the diameter of neural fiber trunks in diagnosing the length of the aganglionic segment. Patients were divided into two groups, “long segment” and “rectosigmoid,” based on the observed length of the affected segment in contrast enema and pathological findings [4]. Subsequently, the diameter of neural fiber trunks was measured, and statistical analysis was performed to evaluate the accuracy of this method. With a cutoff of 40 micrometers, the sensitivity and specificity of this approach were found to be 66.7% and 45.2%(kappa, respectively. Moreover, in this study, the Cohen’s kappa index was calculated to be 0.7, with a p-value of less than 0.001(k = 0.7, p-value < 0.001). These findings suggest that using the diameter of neural fiber trunks to determine the length of the aganglionic segment may not yield high accuracy. In general and based on our study findings, it can be concluded that the diameter of nerve fibers in patients with Hirschsprung’s disease cannot improve the accuracy of measuring the length of the aganglionic segment. Comparing this method with intraoperative biopsy indicates that using nerve fiber diameter yields lower accuracy and reliability during surgery. Therefore, based on our study, the use of nerve fiber diameter is not recommended for determining the length of the aganglionic segment in these patients.

## Limitation

Our report was cross sectional study that might cause a selection bias. Moreover, small number of subjects in our report implies further study with a larger sample size is necessary to clarify and confirm our findings.

## Data Availability

The datasets used and/or analysed during the current study are available from the corresponding author on reasonable request.

## Abbreviations

RSB: Rectal suction biopsy
